# Supplementary material for: Fractional re-distribution among cell motility states during ageing
Source: Commun Biol. 2021 Jan 19;4:81. doi: 10.1038/s42003-020-01605-w (PMC7815872; doi:10.1038/s42003-020-01605-w)
Supplement: Supplementary file 2 — Supplementary Information [file 42003_2020_1605_MOESM2_ESM.pdf]

Supplementary Information

Manuscript Title: Fractional re-distribution among cell motility states during ageing

Authors: Jude M. Phillip, Nahuel Zamponi, Madonna P. Phillip, Jena Daya, Shaun McGovern, Wadsworth Williams, Katherine Tschudi, Hasini Jayatilaka, Pei-Hsun Wu, Jeremy Walston, Denis Wirtz

Supplementary figure 1. Cross correlation analysis of motility parameters with age

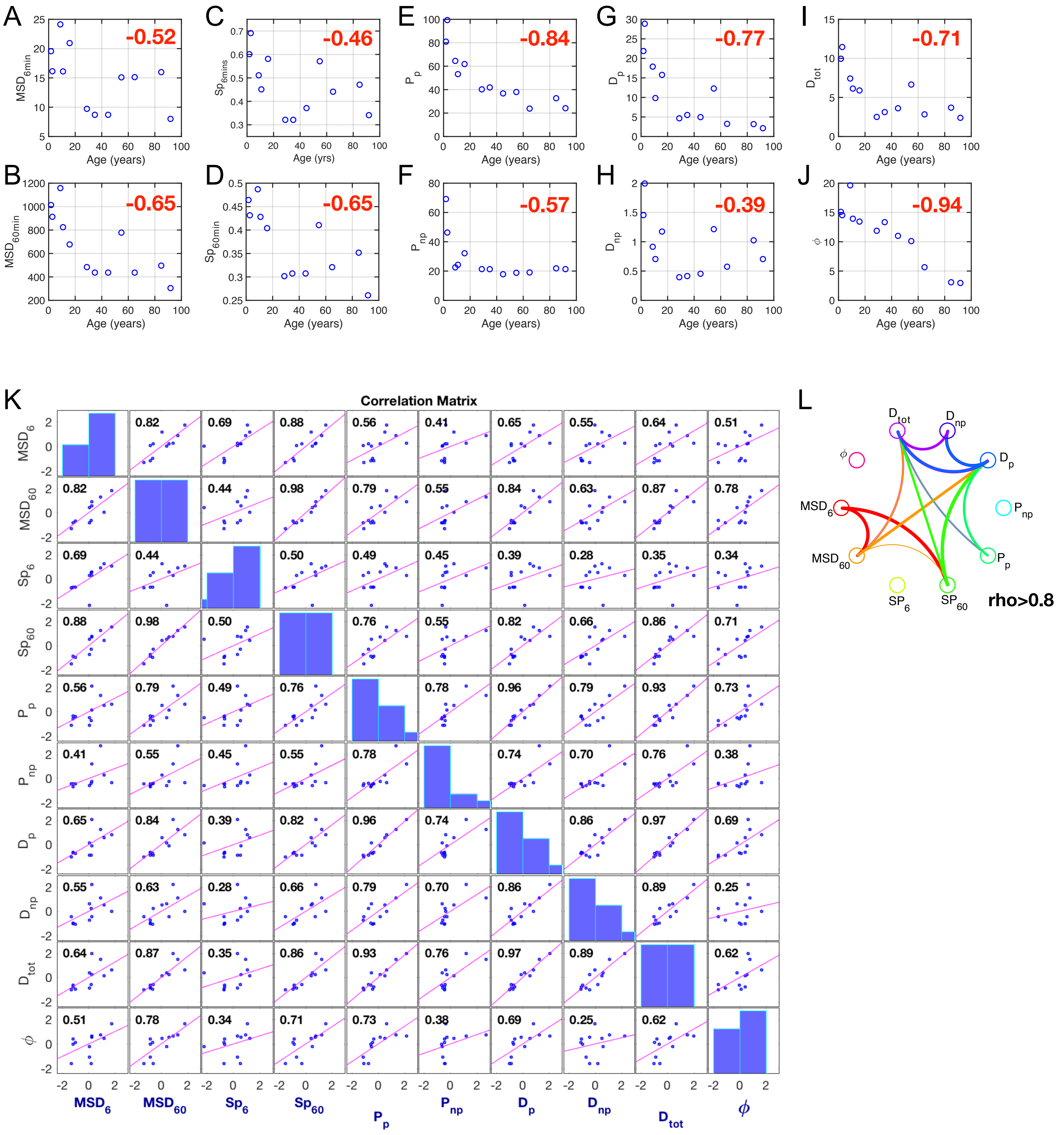

Supplementary Figure 1. Cross correlation analysis of motility parameters with age.

A-J. Pearson correlation analysis of global cell-motility parameters with increasing age; MSD6 (**A**), MSD60 (**B**), SP6 (**C**), SP60 (**D**), Pp (**E**), Pnp (**F**), Dp (**G**), Dnp (**H**), Dtot (**I**), ϕ (**J**). **K.** Cross correlation analysis across parameters with age; each dot represents a donor, with Pearson correlation coefficients denoted in the upper left of the figure. **L**. Circos plot showing the magnitudes of the correlation coefficients among parameters, connected nodes denote cross correlations above 0.8, with the thickness of the lines showing the scaled magnitude.

Supplementary figure 2. Log normalization of cellular motility parameters distributions

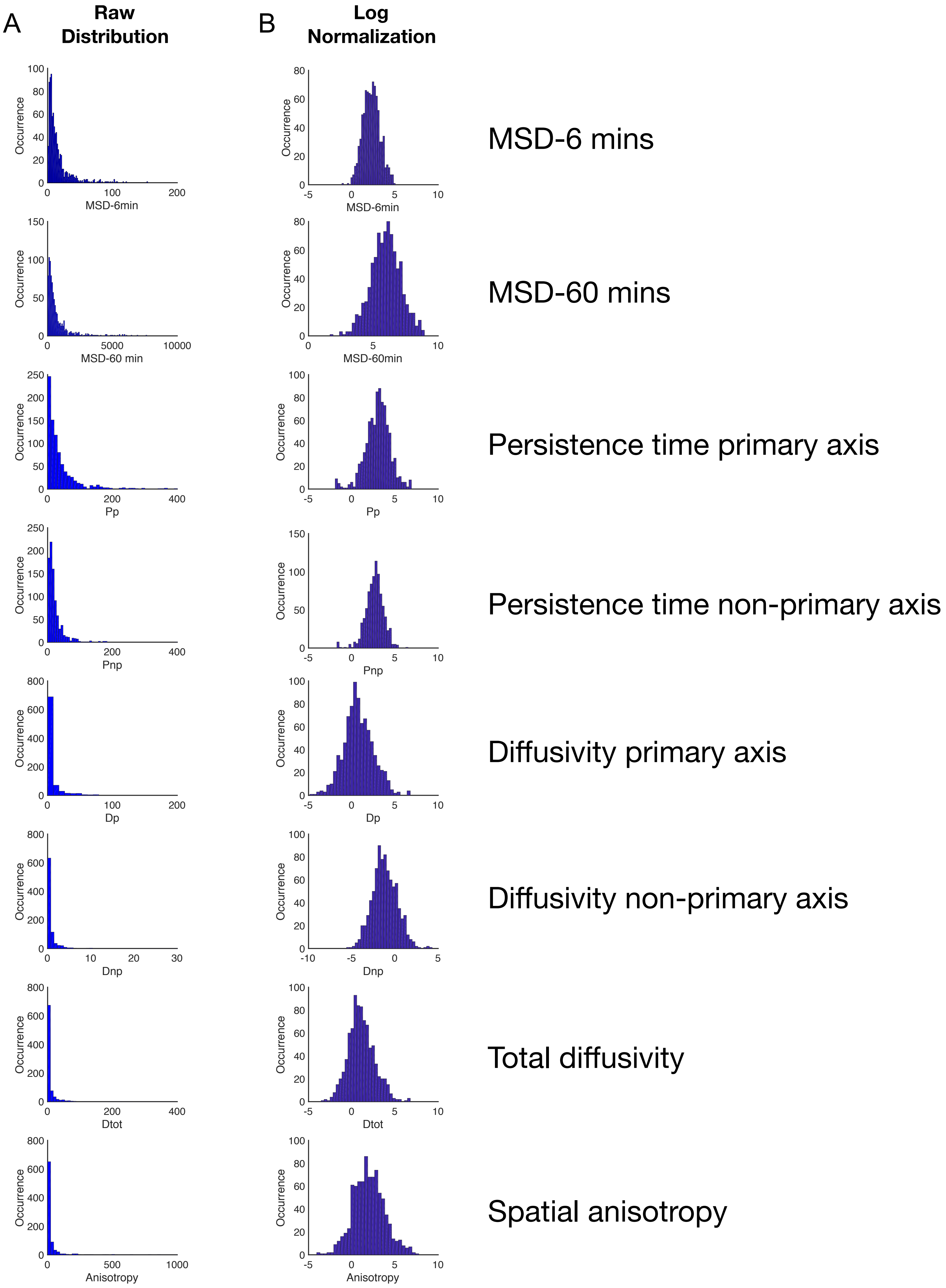

Supplementary Figure 2. Log normalization of cellular motility parameter distributions.

A. Raw distributions of eight motility parameters. **B**. Log normalization of the corresponding distributions for motility parameters.

Supplementary figure 3. Correlation analysis for cellular motility parameters at the single cell level

A

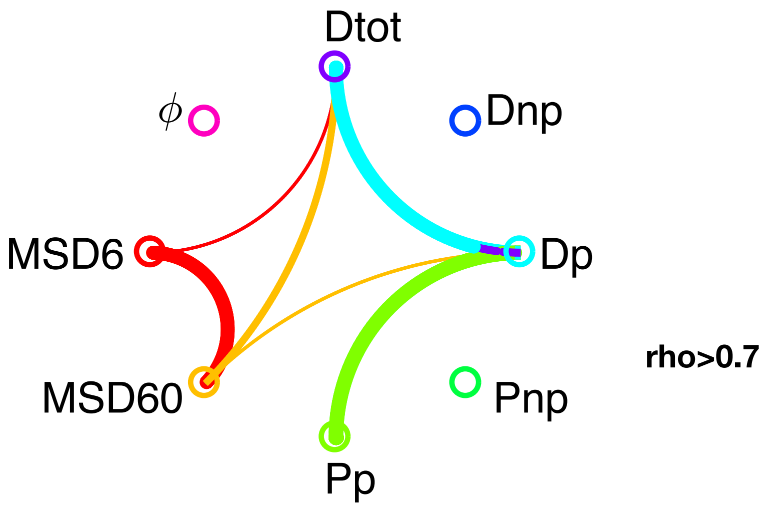

B

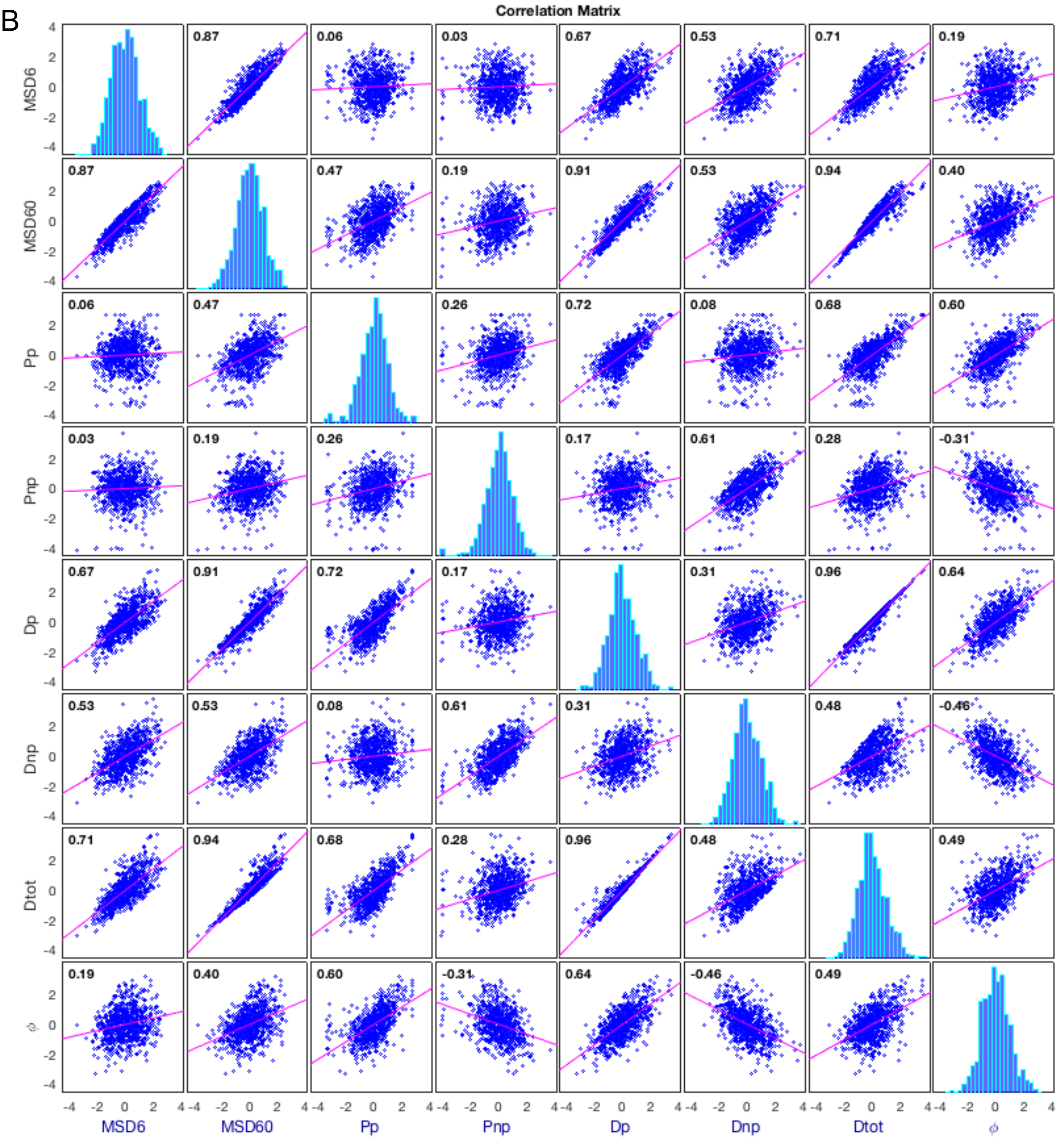

Supplementary Figure 3. Correlation analysis of motility parameters at the single-cell level.

A. Circos plot showing the cross correlation among the eight motility parameters at single cell resolution. Lines connecting nodes denote correlation coefficients greater than 0.7. **B.** Cross correlation analysis among eight motility parameters across all ages, each dot represents a single cell, with the linear regression line shown in magenta, and the magnitude of the Pearson correlation coefficient displayed in the upper left corner of the plots.

Supplementary figure 4. Single cell spatial motility clusters are defined by the magnitude of motility parameters

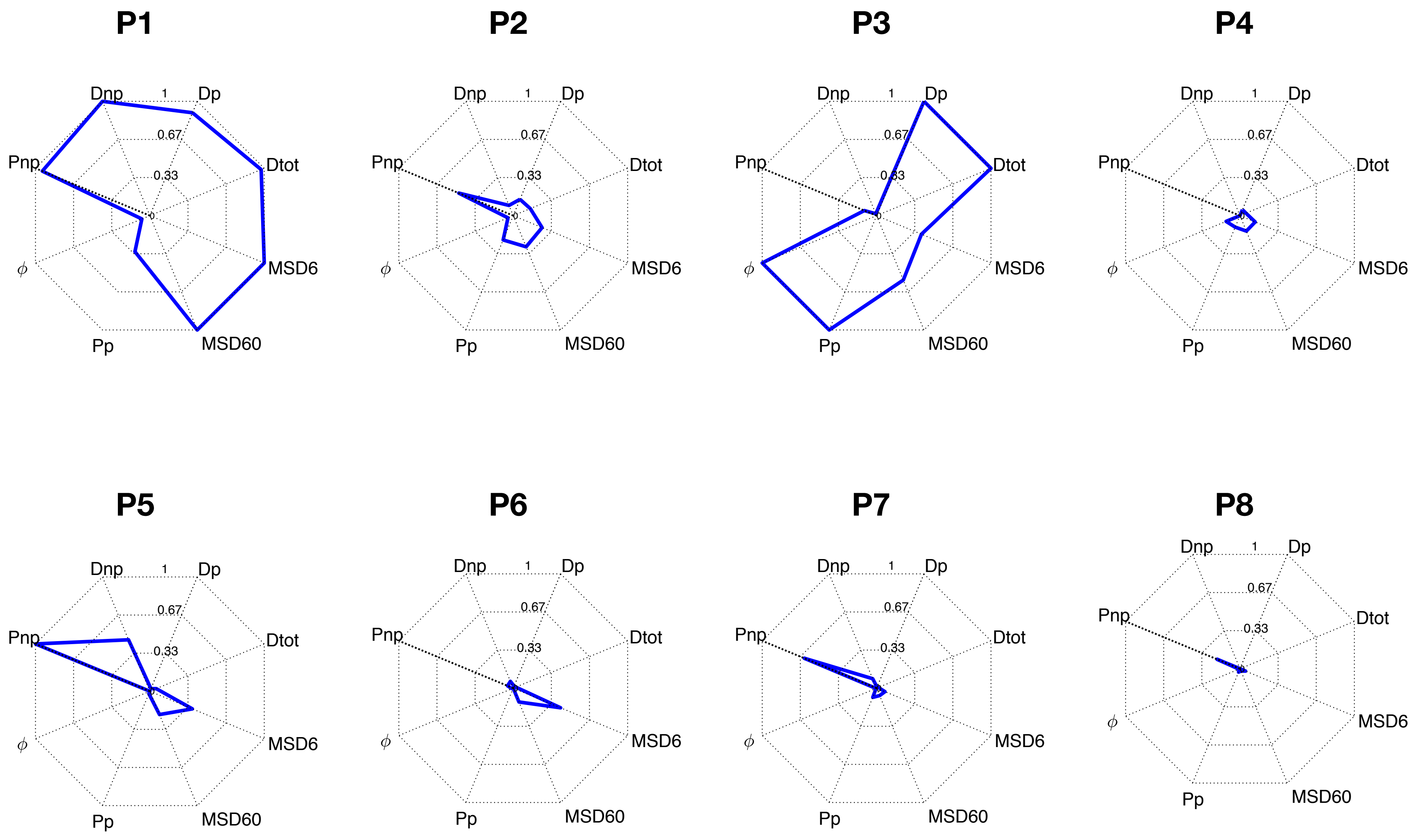

Supplementary Figure 4. Single cell spatial motility clusters are defined by similarities in the magnitude of motility clusters.

A. Radar plots showing the normalized magnitudes (z-score) for the eight motility parameters defining the eight distinct motility clusters.

Supplementary figure 5. Age-dependent fractional distribution of cells within each spatial cluster

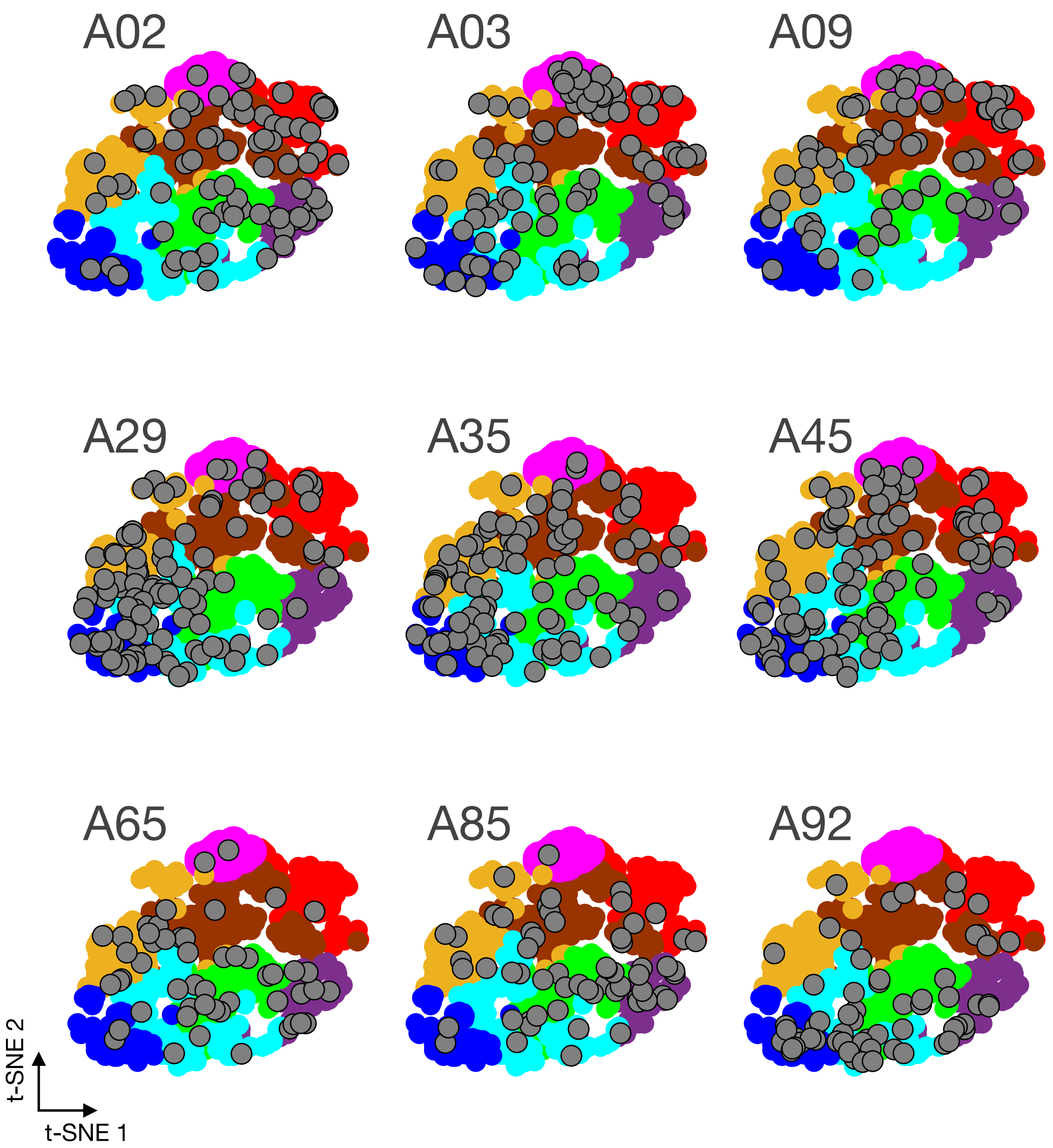

Supplementary Figure 5. Age-dependent fractional distribution of cells within each spatial cluster.

2-Dimensional t-stochastic neighbor embedding plots showing the overlay of cells from each age per spatial cluster (P1-P8)

Supplementary figure 6. Age-dependent motility parameters is approximated as the weighted average per spatial cluster

A

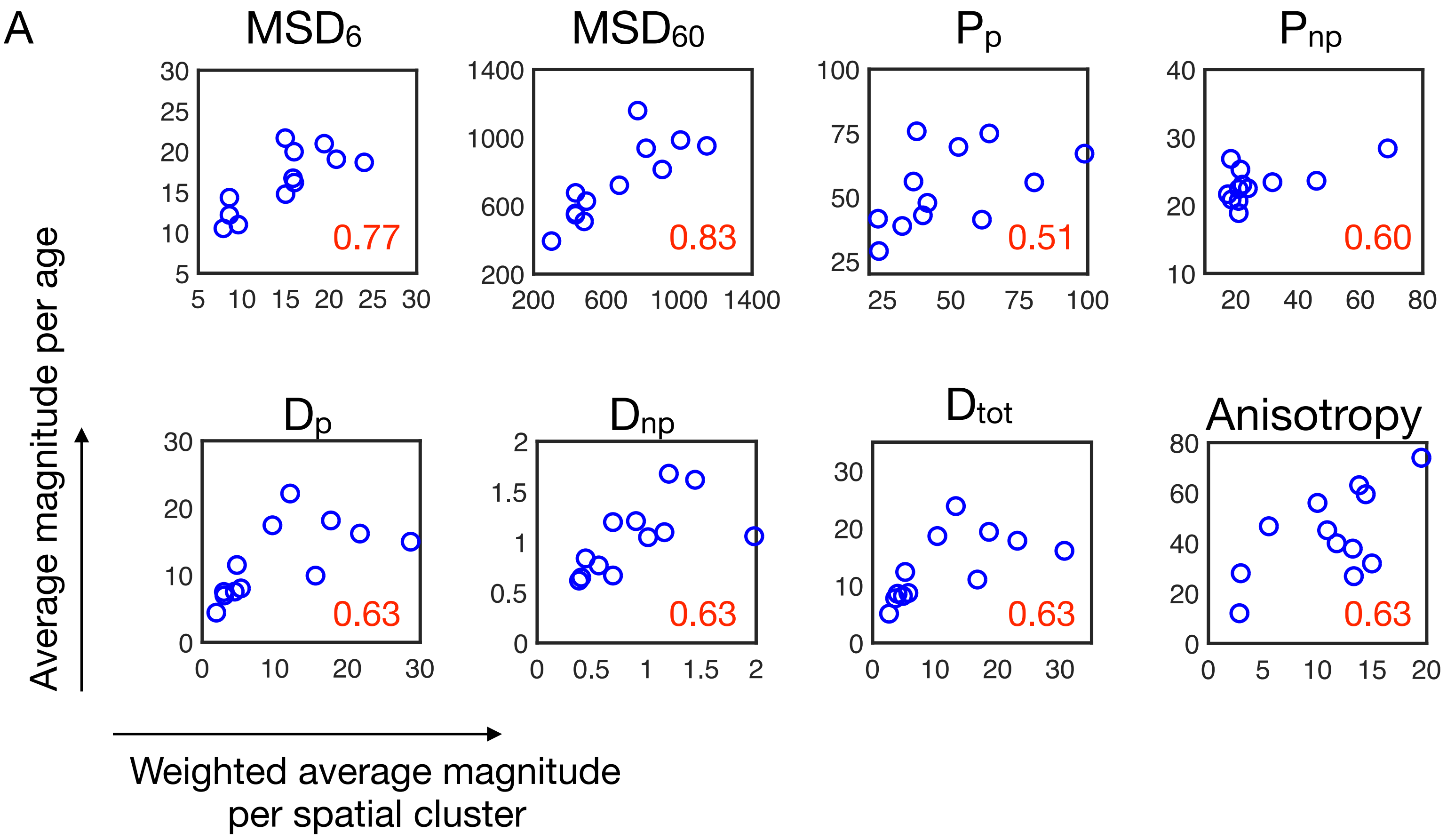

B

	A02	A03	A09	A11	A16	A29	A35	A45	A55	A65	A85	A92
**Overall Correlation**	0.999	0.997	0.998	0.999	0.999	0.998	0.999	0.999	0.999	0.997	0.999	1.000
**Correlation excluding MSD60**	0.812	0.640	0.711	0.738	0.873	0.787	0.871	0.843	0.787	0.528	0.784	0.959

Supplementary Figure 6. Age-associated motility parameters scale as weighted averages per spatial cluster.

A. Scatter plots showing the correlation between the weighted average per spatial cluster and the average magnitude of the parameter cluster. Pearson correlation coefficient is denoted in red in the bottom right side of plots. The weighted average was calculated as the sum of the fractions times the average magnitude per cluster. **B**. Table showing the correlation of the sum of the weighted averages per cluster with all average motility parameters as a function of age.

Supplementary figure 7. Cellular activity based on Point-process analysis.

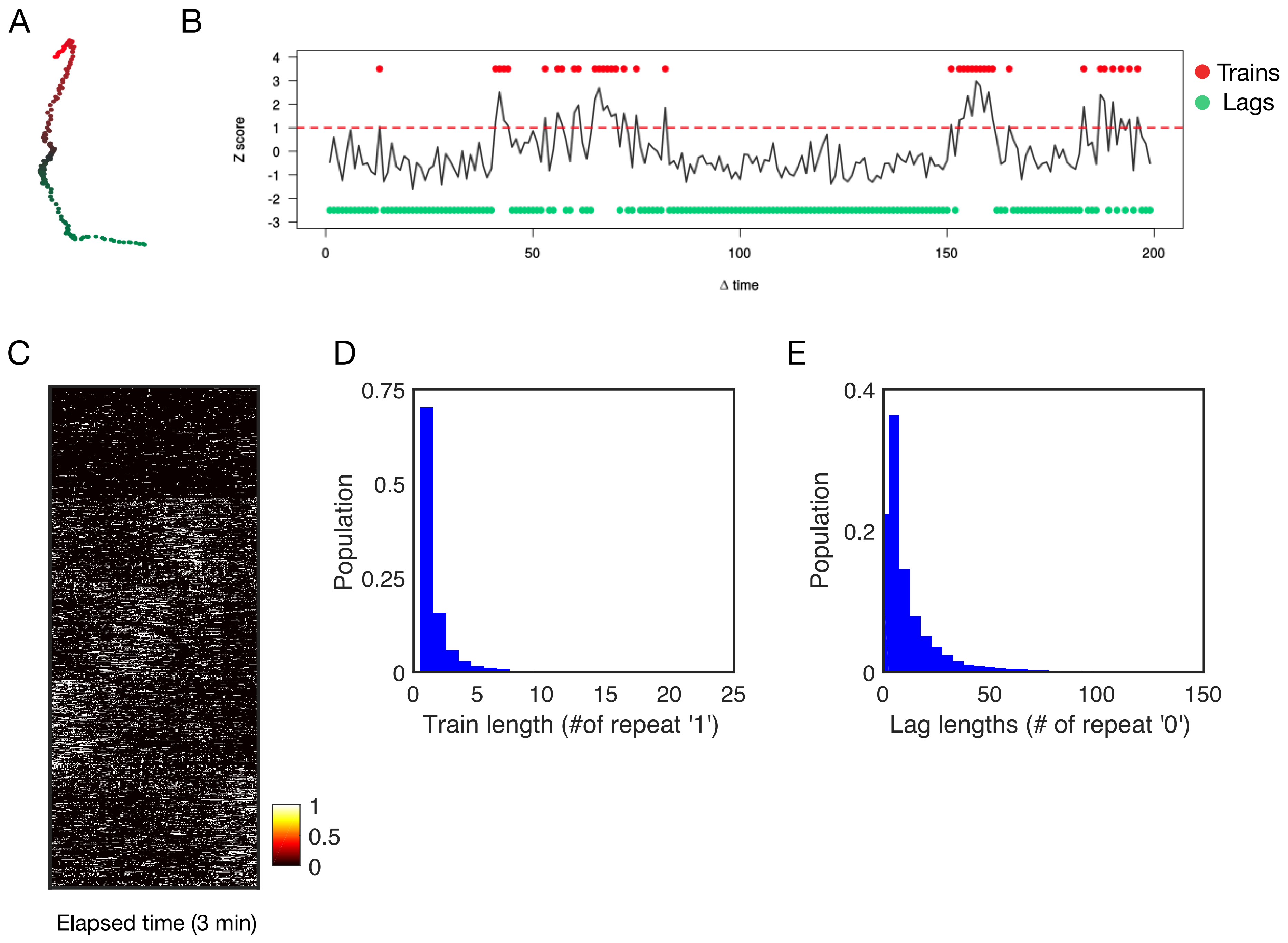

Supplementary Figure 7. Cellular activity based on point-process analysis.

A. x-y trajectory of a single cell. **B.** 1-Dimensional displacement profile for cells shown in (A), highlighting how we convert the raw activity profile is transformed into a binary profile based on a threshold of 1 standard deviation above the baseline. Red dashed line denotes 1SD, with red circles denoting trains and green circles denoting lags. **C**. Heat map showing the binary activity profiles per cell, ordered based on 4 activity groups show in Figure 3C. **D-E**. Distribution of train lengths (D) and lag lengths (E) across all cells for all ages.

Supplementary figure 8. Association among Spatial and Activity clusters per age

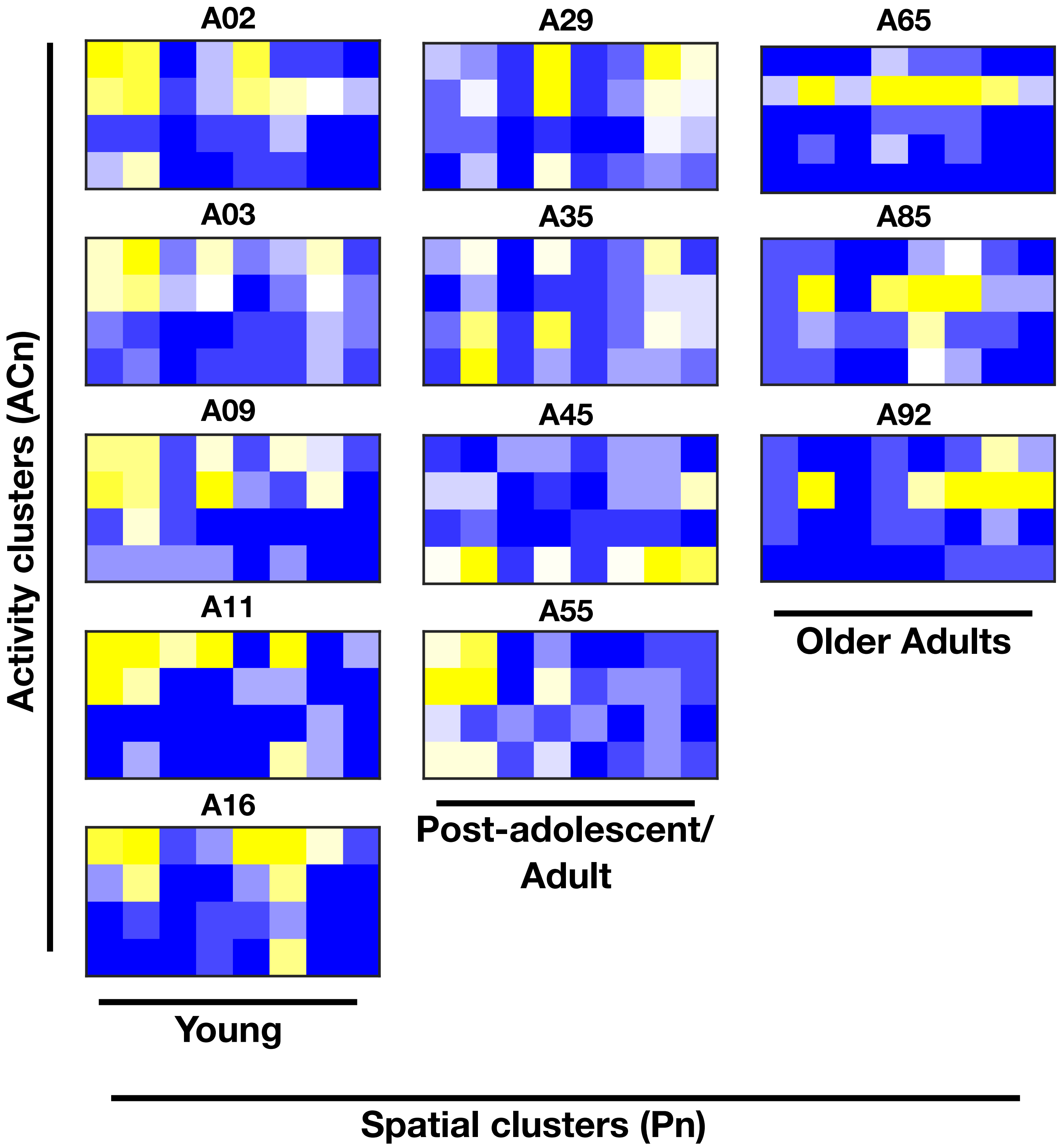

Supplementary Figure 8. Association among spatial and activity clusters per age. Heatmaps showing the frequencies of cells per motility state for all ages. Each abundance is normalized based on the maximum value of frequencies per age.

Supplementary figure 9. Determination of likely progression order among spatial clusters

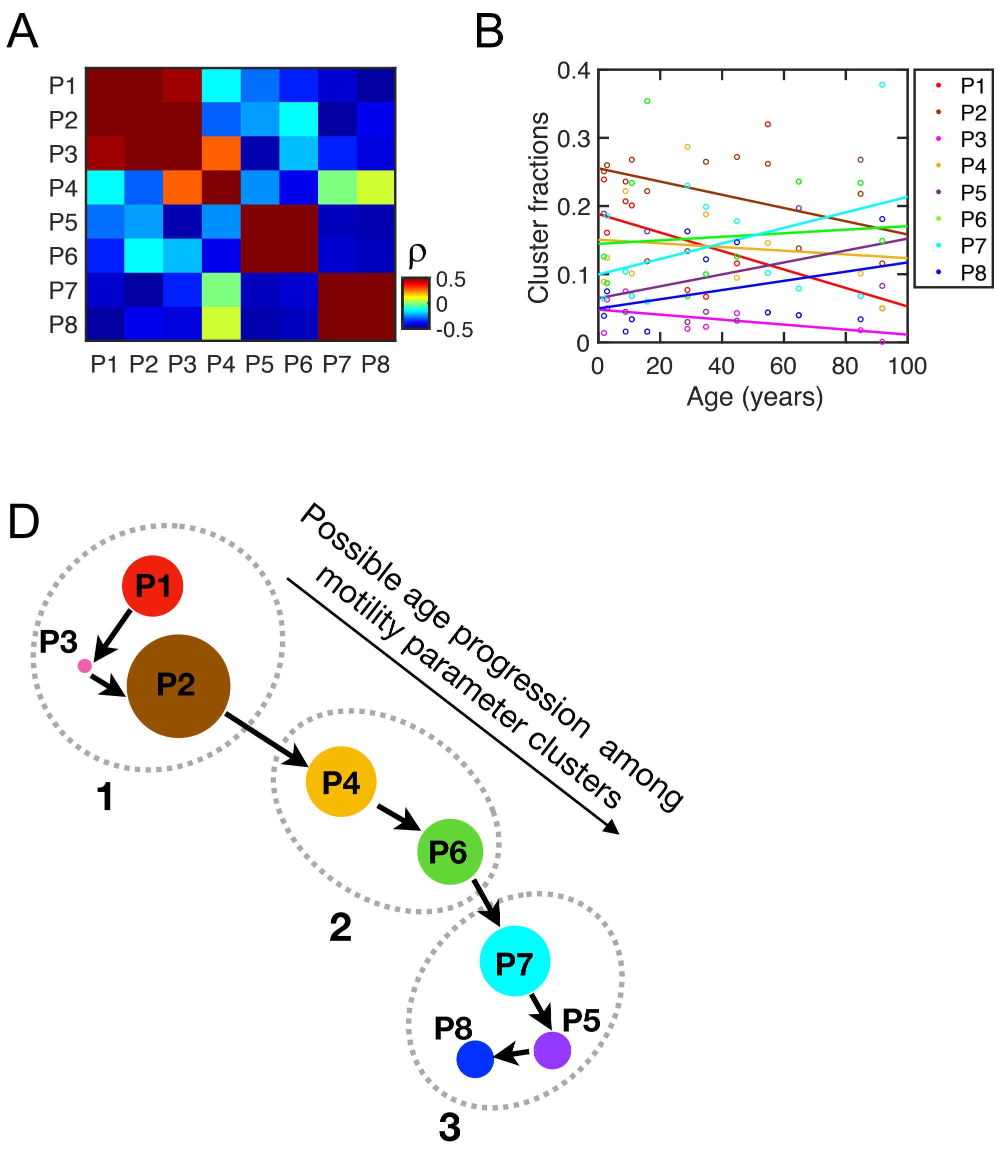

Node size = cells per cluster
Link length = cross-correlation coefficient

Supplementary figure 9. Determination of likely progression order among spatial clusters.

A. Heatmap showing the magnitude of the age correlation among eight clusters. Color scale from blue to maroon denoting low to high, with a range of Pearson correlation coefficients from -0.5 to 0.5. **B**. Pearson correlation trends showing the relationship of cell fraction within each color-coded cluster with increasing age. **C**. Bar plots showing the magnitude of the age correlations for each cluster shown in (B). **D**. Proposed progression order of cells within each cluster with age. Since aging is a continuum, the order was determined based on the magnitude of the correlation for each of the 8 clusters with increasing age, and the associating among clusters (how close or far from each other) was determined based on the cross correlation among clusters across all ages. Results indicate that the likely progression order is P1, P3, P2, P4, P6, P7, P5, P8. **F**. Bar plot showing the proportion of cells being classified in each spatial and activity cluster.
