# Supplementary material for: Fractional re-distribution among cell motility states during ageing
Source: Commun Biol. 2021 Jan 19;4:81. doi: 10.1038/s42003-020-01605-w (PMC7815872; doi:10.1038/s42003-020-01605-w)
Supplement: Supplementary file 3 — Description of Additional Supplementary Files [file 42003_2020_1605_MOESM3_ESM.pdf]

Description of Additional Supplementary Files

File name: Supplementary Data 1.

Description: Name and age for samples used in this study

File name: Supplementary Data 2.

Description: Text file containing the x-y coordinates for all cells analyzed and presented, column 1 denotes the donor ID, column 2 denotes the cell ID, column 3 indicates the tracking frame number (per 2 mins), and column 4 and 5 denotes the x and y coordinates respectively.

File name: Supplementary Data 3.

Description: Mean values and z-scores for the 10 bulk motility parameters per age

File name: Supplementary Data 4.

Description: p-values indicating enrichments and depletions for each spatial cluster per age based on null hypothesis testing.

File name: Supplementary Data 5.

Description: p-values indicating enrichments and depletions for each activity cluster per age based on null hypothesis testing.

File name: Supplementary Data 6.

Description: p-values indicating enrichments and depletions for spatial clusters per activity cluster based on null hypothesis testing.

File name: Supplementary Data 7.

Description: p-values indicating enrichments and depletions for spatial clusters per activity cluster for each age group based on null hypothesis testing.
